# Dynamics of Inflammatory Parameters in Hospitalized and Surgically Treated Patients with Odontogenic Abscesses

**DOI:** 10.3390/medicina62040614

**Published:** 2026-03-24

**Authors:** Dinko Martinovic, Ema Puizina, Boris Kos, Jasna Puizina, Laura Jurina, Lovre Martinovic, Marko Kumric, Daniela Supe Domic, Ivica Luksic, Emil Dediol, Josko Bozic

**Affiliations:** 1 Department of Maxillofacial Surgery, University Hospital of Split, 21000 Split, Croatia; 2 Department of Maxillofacial Surgery, University of Split School of Medicine, 21000 Split, Croatia; 3 Department of Maxillofacial Surgery, Dubrava University Hospital, 10000 Zagreb, Croatia; 4 Department of Biology, Faculty of Science, University of Split, 21000 Split, Croatia; puizina@pmfst.hr; 5 Dental Medical Studies, University of Split School of Medicine, 21000 Split, Croatia; jurinalaura@gmail.com; 6 Medical Studies, University of Split School of Medicine, 21000 Split, Croatia; 7 Department of Pathophysiology, University of Split School of Medicine, 21000 Split, Croatiajosko.bozic@mefst.hr (J.B.); 8 Department of Medical Laboratory Diagnostics, Faculty of Health Sciences, University of Split, 21000 Split, Croatia; 9 Department of Medical Laboratory Diagnostics, University Hospital of Split, 21000 Split, Croatia; 10 School of Medicine, University of Zagreb, 10000 Zagreb, Croatia

**Keywords:** odontogenic abscess, CRP, procalcitonin

## Abstract

*Background and objectives*: Odontogenic abscess represents a serious infection in the head and neck region with the necessity of immediate treatment. Due to the fast pacing and progression, as well possibly severe consequences of this condition, it is important to have a fast and reliable biomarker to adequately monitor these patients. Since serum procalcitonin and C-reactive protein (CRP) are the most commonly used clinical biomarkers to monitor serious infections, the aim of this study was to investigate their temporal profiles in hospitalized patients undergoing surgical management of odontogenic abscesses. *Materials and methods*: This longitudinal, multicentric study was conducted on 65 patients with odontogenic abscesses at the University Hospital of Split and Dubrava University Hospital. Biomarker levels were assessed at admission and at four time points during the early and middle postoperative periods to evaluate initial elevations, treatment-associated changes, and differences in kinetic behavior. *Results*: After converting real procalcitonin and CRP values to proportions, a Δ between the time points was calculated. There was a statistically significant difference in the Δ proportion between procalcitonin and CRP in the 0–6 h time frame (19.3 (10.6–27.8)% vs. 7.2 (−3.0–20.4)%, *p* < 0.001) and the 24–48 h time frame (30.8 (24.5–35.0)% vs. 51.7 (30.5–57.7)%, *p* < 0.001). Furthermore, multiple linear regression analysis showed that procalcitonin at time point 0 (*p* = 0.037), 6 h (*p* = 0.009) and 24 h (*p* = 0.038) significantly predicted hospitalization duration after model adjustment for age, gender, BMI and pre-admission antibiotic treatment. *Conclusions*: The findings of this study show that procalcitonin exhibits a faster and more pronounced decrease in the early postoperative period compared with the CRP values. Following the middle postoperative period both biomarkers decreased in association with clinical improvement; however, procalcitonin demonstrated an earlier and more consistent decline. The observed pattern indicates a rapid dynamic of procalcitonin values during the early postoperative phase and supports its potential value for early monitoring of surgical treatment response in odontogenic abscesses.

## 1. Introduction

Odontogenic abscess represents a localized infectious inflammation and accumulation of purulent material within the head and neck region [1]. The pathogenesis involves bacterial invasion of the dental pulp through extensive dental caries, followed by spread through the root canal system to periapical tissues, and can further progress to life-threatening complications if not recognized and managed promptly [2,3]. The most common etiologic factors include untreated dental caries, failed endodontic treatment, traumatic dental injuries, surgical trauma to the jaws and retained root fragments, with mandibular molars being the predominant source of severe infections that spread into deep fascial spaces of the head and neck [4,5,6]. The primary bacterial cause is usually polymicrobial, but as the infection progresses and anaerobic conditions develop, the bacterial population shifts toward strict anaerobes, which enzymatically degrade tissue-matrix proteins and produce metabolic byproducts that further promote bacterial proliferation [7,8]. When such bacterial infections disseminate and trigger systemic inflammation, acute-phase proteins become critical indicators of disease severity and treatment response [9,10].

C-reactive protein (CRP) is among the most reliable laboratory indicators of infection and systemic inflammation, belonging to the class of acute-phase proteins, whose synthesis in hepatocytes is upregulated by interleukin-6 [11,12]. Following an inflammatory stimulus, serum CRP begins rising within 4 to 6 h, doubling every 8 h and reaching peak concentrations between 36 and 50 h [13]. With a constant half-life of approximately 19 h, CRP kinetics directly reflect the degree and intensity of ongoing tissue damage and inflammatory response [14]. The sensitivity and specificity of CRP in identifying bacterial infections have been well established in hospitalized patients, and its rapid rise, coupled with a subsequent decline during appropriate treatment, makes it a valuable biomarker for the monitoring of therapeutic response [15,16]. However, CRP lacks specificity to discriminate bacterial from viral infections, and its delayed peak response limits early diagnostic utility [17]. Procalcitonin is the precursor peptide of the calcitonin hormone, and it has emerged as a very specific biomarker for systemic bacterial infections [18,19]. Under physiologic conditions, procalcitonin is synthesized almost exclusively in parafollicular cells of the thyroid and rapidly cleaved to mature calcitonin, resulting in minimal circulating procalcitonin levels—typically under 0.02 ng/mL [20,21]. During systemic bacterial infections, however, multiple extrathyroidal tissues, including those of the liver, kidneys, adipose tissue, spleen, and lungs, synthesize procalcitonin via an alternative mechanism, leading to rapid increases in serum concentrations that can rise thousands of times above baseline values [22,23]. Procalcitonin levels begin rising 3 to 4 h following bacterial infection and reach peak concentrations within 12 to 24 h, with a half-life of 22 to 26 h [24].

Despite the well-characterized kinetics of these inflammatory markers in systemic infections, scientific literature specifically investigating their temporal dynamics in hospitalized patients with surgically managed odontogenic abscesses remains limited. Recently, Kim et al. studied 60 patients with odontogenic maxillofacial infections, finding significantly higher mean procalcitonin levels at admission in the sepsis group versus the non-sepsis group, with CRP and white blood cell counts showing no significant differences [25]. Moreover, another recent study found that the Δ of procalcitonin between days 2 and 3 significantly differed in terms of antibiotic appropriateness, with a ≥30% decrease predicting survival in septic patients with odontogenic abscess [26]. However, Urechescu et al. also analyzed maxillofacial infection patients, finding significantly higher CRP levels in patients hospitalized for prolonged periods versus short-stay patients, with an optimal cutoff of 63 mg/L predicting ≥5-day hospitalization [27]. Lastly, a study that evaluated 63 patients with odontogenic abscess recorded procalcitonin levels at presentation, day 4, and day 8 post treatment and showed significantly different values for low-, moderate-, and high-severity infections [28]. Even though these studies showed the significance of both CRP and procalcitonin levels in odontogenic infections and consequent septic states—especially procalcitonin as a great biomarker of sepsis—none measured both procalcitonin and CRP across different time points of odontogenic abscess treatment and compared their temporal dynamics.

Hence, this study aimed to evaluate the temporal changes in serum procalcitonin and CRP levels in hospitalized patients undergoing surgical treatment for odontogenic abscesses, assessing biomarker elevations before surgical management, treatment-related reductions during hospitalization and differences in early and middle postoperative kinetics.

## 2. Materials and Methods

### 2.1. Study Design and Ethical Considerations

This longitudinal prospective study with repeated measurements was conducted during the period from January 2025 to July 2025.

The study was approved by the Ethics Committee of the University Hospital of Split (Number: 2181-147/01-06/LJ.Z.-24-02.) and the Ethics Committee of the Dubrava University Hospital (Number: 2024/0828-1). Furthermore, the study was conducted in accordance with the ethical principles of the 2013 Declaration of Helsinki. Before participating in the research, all participants were informed in detail about the objectives and course of the study and signed an informed consent, which ensured respect for their rights and well-being.

### 2.2. Subjects

The study included subjects who were hospitalized and surgically treated at the Department of Maxillofacial Surgery, University Hospital of Split, Split, Croatia, and the Department of Maxillofacial Surgery, Dubrava University Hospital, Zagreb, Croatia. All subjects underwent surgical treatment (incision and drainage) of odontogenic abscesses in the head and neck area, and the treatment was carried out according to the standards of the medical profession. Participation in the study was completely voluntary and did not affect the course or quality of treatment.

The criteria for inclusion in the study were: age between 18 and 80 years, diagnostically confirmed dental pathology as the cause of the abscess, and surgical treatment of the abscess.

The criteria for exclusion were: active malignant diseases, diabetes, autoimmune diseases, chronic liver or kidney diseases, immunosuppressive therapy, pregnancy, and abuse of psychoactive substances and/or alcohol.

After screening, 20 patients from the University Hospital of Split and 45 patients from the Dubrava University Hospital were included in the study.

### 2.3. Clinical and Laboratory Evaluation

All demographic and other relevant medical data were taken from the anamnesis of the included subjects or extracted from the Integrated Hospital Information System (iBIS). All data were collected and analyzed with strict protection of the subjects’ privacy and confidentiality in accordance with the European Union’s General Data Protection Regulation (EU GDPR, Regulation (EU) 2016/679).

Body mass index (BMI) was determined according to the formula expressed as [BMI = kg/m^2^]. Diagnosis of odontogenic abscess was made by clinical and radiological examination. Multislice computed tomography (MSCT) with intravenous contrast was used to confirm the abscess collection in the head and neck region, and multiplanar reconstruction (MPR) was used to estimate the measures of the collection in all three dimensions, from which the volume was calculated. Furthermore, a microbiological swab was used during the surgical intervention to determine the bacterial pathogen, thereby ensuring diagnostic accuracy.

Laboratory evaluation included venous blood sampling from the cubital vein immediately before surgical incision and drainage of the abscess. The samples were analyzed for inflammatory biomarkers (CRP and procalcitonin), as well as standard laboratory panels (complete blood count, urea, creatinine, liver transaminases, and coagulogram). All laboratory parameters were determined by an experienced medical biochemist according to standard laboratory protocols.

CRP was determined by a turbidimetric immunoassay on a Cobas c703 instrument (Roche, Basel, Switzerland) using the Tina-quant C-Reactive Protein IV CRP4 reagent. The assay demonstrates linearity up to 400 mg/L, precision with CV < 10% and a reference interval < 5 mg/L. Procalcitonin was measured by electrochemiluminescence immunoassay (ECLIA) on a Cobas e801 instrument (Roche, Basel, Switzerland) using Elecsys BRAHMS PCT reagent. The assay shows linearity up to 100 ng/mL, precision with CV < 10% and a reference interval < 0.1 ng/mL. CRP and procalcitonin measurements, except for the initial sample immediately before surgery (0 measurement time point), were repeated 6 h (1st measurement time point), 24 h (2nd measurement time point), 48 h (3rd measurement time point) and 96 h (4th measurement time point) after the surgical intervention, which allowed for monitoring of the dynamics of the inflammatory response over time. These time points were chosen according to the most expected shifts and biomarker kinetics.

### 2.4. Statistical Analyses

The collected data were analyzed using the MedCalc statistical program for Windows^®^ (MedCalc Software, Ostend, Belgium, version 23.4.8). Categorical data are presented as whole numbers (N) and percentages (%), while quantitative data are presented as mean ± standard deviation or median (interquartile range), depending on the normality of the distribution. The normality of the data distribution was assessed using the Kolmogorov–Smirnov test. The chi-square (χ^2^) test was used to analyze categorical variables. Comparisons of serum procalcitonin and CRP levels over 5 time points were assessed using ANOVA for repeated measures with Bonferroni correction. In order to determine the difference in dynamics between CRP and procalcitonin and taking into account their different values, ranges, and measurement units, all values were converted to a proportion (percentage), with the 0 measurement point set as 100%. Proportions of procalcitonin and CRP at time points 1–4 were compared via parametric paired *t*-test. Furthermore, for each subsequent measurement time point, the Δ proportion of change (percentage) was calculated according to the formula expressed as [proportion of change (%) = proportion (%) earlier time point − proportion (%) later time point]. The Δ proportion of change between procalcitonin and CRP was compared using the nonparametric Wilcoxon test. Statistical significance was set at *p* < 0.05 for all comparisons.

## 3. Results

This study included 65 patients with odontogenic abscess who were hospitalized and surgically treated. The median age was 38.0 (27.0–55.0) years, and there were more males (55.4%) than females (44.6%). The mean BMI was 25.6 ± 4.0 kg/m^2^ (Table 1).

Regarding clinical data of the study sample at hospital admission, 27 (41.5%) patients were febrile, and only 8 (12.3%) were not already on antibiotic therapy. The most frequent abscess locations were submandibular (32.3%) and perimandibular (33.8%), while the most common causative teeth were the lower third molar (34.3%) and lower second molar (29.9%) (Table 2).

Furthermore, the median leukocyte count during admission was 12.2 (11.0–17.0) × 10^9^/L, while the median platelet count was 291 (180–358) × 10^9^/L. All hematological and biochemical parameters were inside the typical range (Table 3).

All of the included patients underwent incision and drainage under general anesthesia, out of which 58 (89.2%) were managed with a transcervical approach and 7 (10.8%) were managed with an intraoral approach. The most frequent pathogen according to the microbiology report was *Streptococcus* spp. (48, 54.6%), while the median abscess volume according to MSCT was 25.2 (9.4–62.6) cm^3^. Causative teeth were extracted intraoperatively in 15 (23.3%) patients (Table 4).

**Figure 1:**
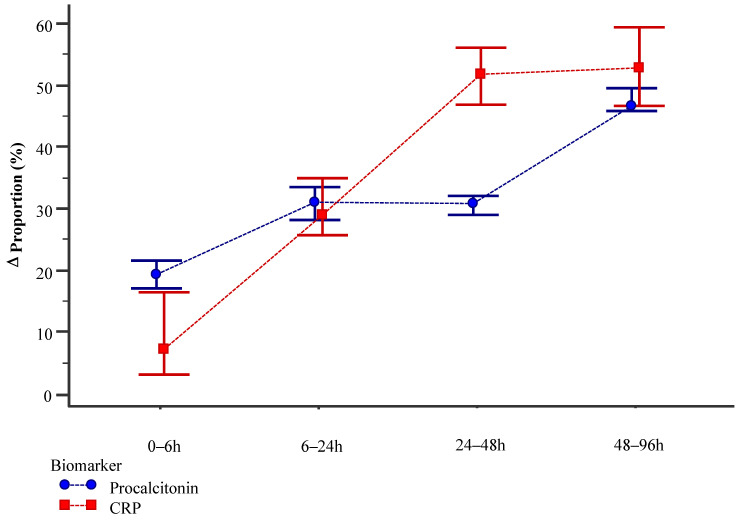
Dynamic of Δ proportion for serum procalcitonin and CRP through 5 time points.

After converting real procalcitonin and CRP values to proportions, a Δ between the time points was calculated. There was a statistically significant difference in the Δ proportion between procalcitonin and CRP in the 0–6 h time frame (19.3 (10.6–27.8)% vs. 7.2 (−3.0–20.4)%, *p* < 0.001) and 24–48 h time frame (30.8 (24.5–35.0)% vs. 51.7 (30.5–57.7)%, *p* < 0.001). Moreover, there was no significant difference in the 6–24 h time frame (31.0 (25.4–42.0)% vs. 29.0 (21.5–41.0)%, *p* = 0.673) and 48–96 h time frame (46.7 (43.8–50.4)% vs. 52.1 (37.5–62.3)%, *p* = 0.168) (Figure 1).

After comparison of procalcitonin values at the five time points, there was a significant difference (*p* < 0.05) between all points, except the time points at 24 h and 48 h. Moreover, in the same comparison for CRP values, there was also a significant difference between all points (*p* < 0.05), except time point 0 and 6 h (Table 5).

Additionally, after converting the real values to proportions (%) and using time point 0 as a referent 100% value, there was a statistically significant difference between procalcitonin and CRP at the 6 h time point (59.5 ± 31.4 vs. 94.4 ± 30.7, *p* < 0.001) and 24 h time point (42.9 ± 28.8 vs. 68.3 ± 29.7, *p* < 0.001) (Table 5).

Multiple linear regression analysis showed that procalcitonin at time point 0 (β ± SE, 3.173 ± 1.543, *p* = 0.037), 6 h (6.894 ± 2.558, *p* = 0.009) and 24 h (9.651 ± 4.774, *p* = 0.038) significantly predicted the duration of hospitalization after model adjustment for age and BMI, with hospitalization duration as a dependent variable (Table 6). The model showed that at these three time points, procalcitonin has a positive predictive value with respect to the duration of hospitalization. The overall regression model was statistically significant (*p* < 0.001), with a coefficient of determination (R^2^) of 0.508 (R^2^ adjusted = 0.370).

## 4. Discussion

The results of this study show that patients with odontogenic abscesses have clearly pronounced differences in the temporal dynamics of serum concentrations of procalcitonin and CRP during the first four days of hospitalization. Already during the first 6 h after admission and surgery, a more pronounced relative decrease in procalcitonin concentration was recorded compared to CRP, which indicates a faster initial reactivity of procalcitonin to surgical treatment. Furthermore, after 24 h, there was a statistically significant median decrease in procalcitonin values of almost 60% compared to the median 30% of CRP decrease, which further establishes procalcitonin as a possibly better marker of early postoperative monitoring in patients with odontogenic abscess. At later time points after surgery, both biomarkers showed a gradual decrease in serum concentrations in parallel with clinical control of the infection, but their relative changes differed depending on the stage of the disease. The decrease in procalcitonin concentration was earlier and more consistent compared to CRP, which showed a more pronounced decrease only in the later stage of recovery. Moreover, multiple linear regression analysis showed that the early procalcitonin values were significant negative predictors of the duration of hospitalization.

As previously mentioned, the observed trend confirms the larger dynamic range of procalcitonin in the acute phase of the disease and suggests its potential as a more sensitive biomarker for early monitoring of therapeutic response in odontogenic abscesses. Numerous studies have evaluated the clinical utility of procalcitonin in the diagnosis and monitoring of systemic bacterial infection [29,30,31]. In contrast, evidence regarding the use of procalcitonin in patients with odontogenic maxillofacial infections remains limited. The clinical study by Kim et al. involving patients with odontogenic maxillofacial infections demonstrated that procalcitonin exhibits higher specificity than CRP and white blood cell count (WBC) in the diagnosis of head and neck odontogenic abscesses and identified procalcitonin as a highly accurate biomarker for the detection of sepsis in this patient population [25]. Comparable findings were reported by Lin et al., who investigated the prognostic value of procalcitonin in elderly patients with severe oral and maxillofacial infections and showed that procalcitonin serves as an early and reliable indicator of disease severity [32]. Furthermore, a recent small prospective study in patients with maxillofacial infections receiving antibiotic therapy demonstrated that procalcitonin was superior to conventional inflammatory markers, including WBC and CRP, for the monitoring of treatment response [33]. Collectively, these studies support the measurement of procalcitonin in patients with odontogenic maxillofacial infections as a rapid and reliable marker of systemic bacterial involvement.

Monitoring of the temporal behavior of serum procalcitonin and CRP in patients with odontogenic abscesses is clinically relevant for optimization of both surgical and antimicrobial therapy—in the latter case, especially in terms of reducing unnecessary antibiotic exposure without adversely affecting outcomes [34,35]. Owing to its high specificity for bacterial infections and rapid response in the acute phase, procalcitonin has been proposed as a valuable biomarker in emergency odontogenic conditions and for early sepsis detection [36,37]. Meta-analytical evidence further supports the superior diagnostic accuracy of procalcitonin over CRP in bacterial sepsis and highlights the benefits of procalcitonin-guided antimicrobial management, including shorter antibiotic courses and reduced mortality [38,39]. In patients with odontogenic and maxillofacial infections, prior studies have reported significantly higher procalcitonin levels at admission in septic compared with non-septic cases, demonstrated the prognostic relevance of early reductions in procalcitonin during therapy, and identified associations of both procalcitonin and CRP concentrations with disease severity and length of hospitalization [25,26,27,28,32]. However, despite the demonstrated relevance of these inflammatory markers, existing studies have largely evaluated procalcitonin or CRP in isolation and at limited time points. In contrast, the present study simultaneously assessed both biomarkers longitudinally, allowing for direct comparison of their temporal dynamics during the surgical and postoperative management of odontogenic abscesses. Moreover, one of the advantages of procalcitonin is that, unlike CRP and other inflammatory markers, procalcitonin production is largely unaffected by nonsteroidal anti-inflammatory drugs (NSAIDs) or corticosteroids and does not rely on white blood cells, remaining a reliable indicator of systemic bacterial infection when anti-inflammatory drugs alter temperature and leukocyte counts [40]. Nevertheless, it should be noted that the serum procalcitonin concentration has been shown, in some studies, to be a less reliable inflammatory marker in infections limited to the orofacial area without systemic complications. Furthermore, procalcitonin levels can rise nonspecifically, even without bacterial infection, in conditions of severe physiological stress, such as major trauma, postoperative states or cardiogenic shock. Additionally, procalcitonin levels remain low in gout, pseudogout, rheumatoid arthritis and some other conditions [40]. That is why some authors advocate for the combined use of CRP, procalcitonin, and clinical status when making decisions about therapy and hospitalization [26,27,28]. Recently, presepsin (sCD14 ST) has been proposed as a new promising biomarker for the early detection of sepsis, rising within 2–3 h and demonstrating high accuracy in distinguishing bacterial infections from non-infectious systemic inflammation [41]. In bacterial infections such as odontogenic abscesses, these biomarkers can complement each other, with procalcitonin and presepsin enabling early detection and CRP reflecting the overall inflammatory response over time [42]. However, further studies are needed regarding this issue to show its clinical utility.

It is also important to highlight that most previous studies investigating the diagnostic relevance of the two inflammatory biomarkers—procalcitonin and CRP—in odontogenic maxillofacial infections have been based on heterogeneous patient cohorts. These studies often include a broad spectrum of cervicofacial infections with varying severity, etiology, and disease course. In several reports, infections of dental origin are not clearly differentiated from other causes (such as tonsillopharyngeal infections, lymphadenitis, or sialadenitis), which limits the direct applicability of the findings to odontogenic abscesses. Furthermore, the lack of standardization in defining “infection severity” hampers meaningful comparisons between studies. Some authors rely on subjective clinical criteria, whereas others incorporate laboratory parameters, comorbidities, or radiological findings, resulting in considerable variability in conclusions regarding the predictive value of these biomarkers. In addition, the timing of sample collection and the frequency of laboratory measurements are not standardized across most studies. Analyses are often limited to comparisons between only two time points (admission and 24 h), without capturing the full temporal dynamics of biomarker changes during treatment. Moreover, biomarker levels, themselves, may vary depending on abscess localization and volume, as well as the patient’s immunological status, further complicating data interpretation. Collectively, these factors substantially limit interstudy comparability and hinder the ability to draw robust conclusions regarding the clinical value of monitoring procalcitonin and CRP dynamics in odontogenic abscesses.

Certain limitations of the present study should also be acknowledged. Even though the study is longitudinal and multicentric, it was still conducted with a relatively small sample size. Moreover, it was impossible to exclude all of the possible confounding effects; for example, all participants were Caucasians, which limited demographic diversity and may restrict the generalizability of the results. Furthermore, most of the included patients were using antibiotic therapy prior to admission, which could potentially interfere with the dynamics of the inflammatory biomarkers. However, we were unable to exclude this bias due to the fact that most patients with odontogenic abscess are initially treated by their primary dentist and antibiotics are the first line of therapy. Lastly, none of the included patients had a severe progression to conditions such as sepsis, necrotizing fasciitis or descending necrotizing mediastinitis; hence, the study is missing data regarding these life-threatening progressions.

## 5. Conclusions

In conclusion, the findings of this study show that procalcitonin exhibited a more rapid and more pronounced decrease in the early postoperative period, reflecting faster responsiveness compared with the CRP values. Following the middle postoperative period, both biomarkers decreased in association with clinical improvement; however, procalcitonin demonstrated an earlier and more consistent decline, whereas CRP showed a more substantial reduction at later stages of recovery. These findings imply that procalcitonin values could possibly be a more valuable tool for the monitoring of early and middle postoperative response to the surgical management of odontogenic abscess.

## Figures and Tables

**Table 1:** Sociodemographic and anthropometric data of the study sample.

Parameter	Study Sample (N = 65)
Age (years)	38.0 (27.0–55.0)
Gender (N, %)	
Male	36 (55.4)
Female	29 (44.6)
Body mass (kg)	76.7 ± 16.0
Body height (cm)	172.1 ± 9.3
BMI (kg/m^2^)	25.6 ± 4.0

All variables are presented as whole numbers (percentage), mean ± standard deviation or median (interquartile range). Abbreviations: BMI—body mass index.

**Table 2:** Clinical data of the study sample during admission.

Parameter	Study Sample (N = 65)
Febrility (N, %)	27 (41.5)
Previous antibiotic therapy (N, %) *	
Amoxicillin + CA	47 (72.3)
Metronidazole	36 (55.4)
Clindamycin	8 (12.3)
None	8 (12.3)
Abscess location (N, %)	
Submandibular	21 (32.3)
Perimandibular	22 (33.8)
Pterygomandibular	16 (24.6)
Buccal	3 (4.6)
Submental	3 (4.6)
Causative teeth (N, %) ^†^	
Lower third molar	23 (34.3)
Lower second molar	20 (29.9)
Lower first molar	16 (23.9)
Lower second premolar	2 (2.9)
Lower central/lateral incisive	3 (4.5)
Upper second premolar	3 (4.5)

All variables are presented as whole numbers (percentage). Abbreviation: CA—clavulanic acid. * some patients received both Amoxicillin + CA and Metronidazole. ^†^ several patients had more than one causative tooth.

**Table 3:** Laboratory parameters during admission.

Parameter	Study Sample (N = 65)
Leukocytes (×10^9^/L)	12.2 (11.0–17.0)
Erythrocytes (×10^9^/L)	4.7 (4.4–5.0)
Platelets (×10^9^/L)	291 (180–358)
Hemoglobin (g/L)	140.2 ± 13.0
Urea (mmol/L)	5.0 (4.0–6.3)
Creatinine (μmol/L)	74.5 (66.0–83.0)
PT (s)	94.7 ± 9.9
APTT (s)	23.0 (22.5–25.0)
ALT (U/L)	22.0 (18.0–30.0)
AST (U/L)	25.1 ± 9.1

All variables are presented as mean ± standard deviation or median (interquartile range). Abbreviations: PT—prothrombin time; APTT—activated partial thromboplastin time; AST—aspartate aminotransferase; ALT—alanine amino transaminase.

**Table 4:** Clinical and surgical data during hospitalization.

Parameter	Study Sample (N = 65)
Incision and drainage during GA (N, %)	65 (100)
Transcervical approach (N, %)	58 (89.2)
Intraoral approach (N, %)	7 (10.8)
Causative tooth extraction (N, %)	15 (23.3)
Surgical drainage tape (N, %)	65 (100)
Microbiology swab (N, %)	65 (100)
Pathogen according to MB report (N, %) *	
*Streptococcus* spp.	94 (54.6)
*Staphylococcus* spp.	43 (25.0)
Other	35 (20.4)
Abscess volume according to MSCT (cm^3^)	25.2 (9.4–62.6)
Hospitalization duration (day)	6.2 ± 1.8

All variables are presented as whole numbers (percentage), mean ± standard deviation or median (interquartile range). Abbreviations: GA—general anesthesia; MB—microbiology; MSCT—multislice computed tomography. * Most patients had a polymicrobial microbiology report.

**Table 5:** Dynamics of serum procalcitonin and hs-CRP concentration in the study sample (N = 65).

	Parameter	Time Points					*p* *
		0	6 h	24 h	48 h	96 h	
Real value	PCT ng/mL	0.59 (0.42–1.12)	0.42 (0.33–0.60)	0.27 (0.23–0.34)	0.25 (0.22–0.31)	0.03 (0.12–0.16)	<0.001 ^a^
	CRP mg/L	92 (48–177)	92 (43–181)	69.1 (25.4–143.0)	29.2 (14.8–64.5)	12.5 (7.3–30.4)	<0.001 ^b^
Proportion (%)	PCT	100	59.5 ± 31.4	42.9 ± 28.8	38.2 ± 25.0	22.8 ± 18.7	/
	CRP	100	94.4 ± 30.7	68.3 ± 29.7	36.6 ± 20.9	18.4 ± 12.8	/
	*p* ^†^	/	<0.001	<0.001	0.692	0.127	

All variables are presented as mean ± standard deviation or median (interquartile range). Abbreviations: PCT—procalcitonin; CRP—C-reactive protein. * ANOVA for repeated measures with Bonferroni correction. ^†^ paired *t*-test. ^a^ All groups except 24 h and 48 h have a statistically significant difference (*p* < 0.05). ^b^ All groups except 0 and 6 h have a statistically significant difference (*p* < 0.05).

**Table 6:** Multiple linear regression analysis for independent predictors of hospitalization duration (days).

Parameter	β *	SE	t	*p*
Age (years)	0.006	0.015	0.391	0.697
BMI (kg/m^2^)	0.117	0.062	1.888	0.064
Female gender ^†^	−0.756	0.479	−1.580	0.120
No pre-admission antibiotic treatment ^‡^	−0.251	0.742	−0.339	0.735
CRP 0 (mg/L)	0.012	0.008	1.590	0.118
CRP 6 h (mg/L)	0.016	0.012	1.373	0.175
CRP 24 h (mg/L)	0.020	0.014	1.449	0.153
CRP 48 h (mg/L)	0.042	0.027	1.554	0.126
CRP 96 h (mg/L)	0.014	0.030	0.485	0.629
PCT 0 (ng/mL)	3.173	1.543	2.056	0.037
PCT 6 h (ng/mL)	6.894	2.558	2.695	0.009
PCT 24 h (ng/mL)	9.651	4.774	2.021	0.038
PCT 48 h (ng/mL)	10.717	8.964	1.196	0.237
PCT 96 h (ng/mL)	3.337	6.809	0.490	0.626

Abbreviations: PCT—procalcitonin; CRP—C-reactive protein; SE—standard error. ^†^ Male gender is the reference group. ^‡^ Pre-admission antibiotic treatment is the reference group. * Unstandardized coefficient (β).

## Data Availability

All data is available upon request to the corresponding author.
